# Cell Lines of Circulating Tumor Cells: What Is Known and What Needs to Be Resolved

**DOI:** 10.3390/jpm12050666

**Published:** 2022-04-21

**Authors:** Yutaka Shimada, Tetsuo Sudo, Shusuke Akamatsu, Takuro Sunada, Akira Myomoto, Kiyoshi Okano, Kazuharu Shimizu

**Affiliations:** 1 Department of Nanobio Drug Discovery, Graduated School of Pharmaceutical Sciences, Kyoto University, Kyoto 606-8501, Japan; sudo.tetsuo.7e@kyoto-u.ac.jp (T.S.); myomoto.akira.6f@kyoto-u.ac.jp (A.M.); okano.kiyoshi.4m@kyoto-u.ac.jp (K.O.); kz.shmz@gmail.com (K.S.); 2 Department of Urology, Graduate School of Medicine, Kyoto University, Kyoto 606-8507, Japan; akamats@kuhp.kyoto-u.ac.jp (S.A.); suna100@kuhp.kyoto-u.ac.jp (T.S.)

**Keywords:** cell line, circulating tumor cell, long-term culture, short-term culture

## Abstract

The importance of circulating tumor cells (CTC) is well recognized. However, the biological characteristics of CTC in the bloodstream have not yet been examined in detail, due to the limited number of CTC cell lines currently available. Thirty-nine CTC cell lines were reported by 2021. For successful cell culturing, these CTC cell lines were reviewed. Previous studies on short-term cultures of CTC also analyzed approaches for establishing the long-term culture of CTC. Negative selection, hypoxic conditions, three-dimensional conditions, and careful management are preferable for the long-term culture of CTC. However, the establishment of CTC cell lines is dependent on the specific characteristics of each cell type. Therefore, a method to establish CTC cell lines has not yet been developed. Further efforts are needed to resolve this issue.

## 1. Introduction

### 1.1. Recent Issues in Circulating Tumor Cell Research

The importance of circulating tumor cells (CTC) has recently been recognized. Although the cultivation of CTC is very promising as a preclinical model for human cancer, difficulties are associated with establishing CTC cell lines due to the low number of CTC in blood and the majority of these cells not being viable. Only a limited number of permanent cell lines of CTC are currently available and their management is more challenging than that of standard cell lines.

CTC cell lines are important for examining the biology of CTC, but not for providing quick feedback to cancer patients. Furthermore, the translational relevance of cell lines is often questioned because prolonged cultures and multiple passages lead to phenotypes that are no longer representative of the original tumor in terms of cell epigenetics and gene expression profiles [1]. Therefore, the focus of research has recently shifted to the clinical use of short-term cultures of CTC. Therefore, the biology of CTC has not yet been investigated in detail.

### 1.2. Our Previous Experience

We previously established cancer cell lines from several tissue specimens, including esophageal and pancreatic cancer [2,3]. We identified monolayer epithelial growth potential and cell line establishment capability as significant prognostic factors in patients with esophageal squamous cell cancer (ESCC) [4,5].

The systematic detection of CTC was initially reported by other research groups. Racila et al. [6] used an immunomagnetic separation and flow cytometry protocol that relied on epithelial cell adhesion molecule (EpCAM)-positive expression. We subsequently developed a short-term culture of CTC using the same type of bead selection method and demonstrated that the colonies that formed contained a slightly higher number of CTC [7].

The majority of conventional cancer cell lines generally proliferate on a two-dimensional (2D) environment, in contrast to the 3D environment of most CTC cell lines, such as spheroids or organoids. We established a small cell esophageal cancer cell line (TYUC-1) that actively grew with sphere formation in a standard culture flask from a tissue specimen [8]. This experience prompted us to establish CTC cell lines.

### 1.3. Aim of This Review

Concerning the approaches to long-term culture, we also examined the literature for short-term cultures. Our aim was to obtain a more detailed understanding of the characteristics of CTC and several methods that support the establishment of long-term cultures from short-term cultures. Based on previous findings and our own research, we herein discussed critical issues facing the development of CTC culture methodologies and proposed a rational strategy for the long-term culture of CTC.

In this review, a long-term culture (CTC cell line) is defined as a culture period of longer than 1 year or continuous passages for more than 6 months. A short-term culture is defined as a culture period of longer than 1 week. We briefly discussed culture-related factors, such as methods for enriching CTC, the characteristics of CTC in the bloodstream, and stem cell biology. We excluded methods for ultra-short term (less than 1 week) cultures and expression assays because the focus of these methods is clinical evaluation, not long-term cultures. We mainly concentrated on the development of a reproducible protocol for establishing CTC cell lines. We also excluded details on the CTC capture system, clinical applications, genetic analyses, CTC-derived xenografts, and cultures of bone marrow disseminated tumor cells.

## 2. Long-Term Culture of CTC

### 2.1. Overview

Thirty-nine CTC cell lines were reported by 2021: 11 from colon cancer, 13 from breast cancer, 2 from prostate cancer, 2 from gastroesophageal cancer, 5 from small cell lung cancer, 2 from non-small cell lung cancer, and 4 from malignant melanoma. Establishment rates ranged between 1 and 17%, with most studies achieving a rate <10% (Table 1 [9,10,11,12,13,14,15,16,17,18,19,20,21,22,23,24,25]).

Several CTC cell lines were established from the same patients at different time points during their follow-up [17,19]. Therefore, these patients had specific CTC that were adapted for in vitro culture conditions. Cayrefourcq et al. [12] reported that two spheroids from the same cell line exhibited different characteristics, which revealed the non-uniform characteristics of CTC.

Epithelial–mesenchymal transition (EMT) cells should survive in the blood because they negligibly express EpCAM. Therefore, EpCAM is not a standard marker for the establishment of cell lines. However, some cells express EpCAM and maintain epithelial characteristics. Additionally, many cells express stem cell markers, such as CD133, CD44, CD24, and ALDH1. These findings indicate that CTC have a mixed epithelial phenotype, an intermediate epithelial/mesenchymal phenotype, and stem cell-like properties.

Stem cell concepts have been employed for long-term CTC cultures. CTC as non-adherent spheres may reflect the intrinsic properties of cancer stem cells that remain viable in the bloodstream after the loss of their attachment to the basement membrane [26]. However, CTC with these stem cell features have not yet been isolated for CTC cultures. We were also unable to establish a primary culture of sorted stem marker (p75NTR+) CTC from 23 patients [27].

### 2.2. Culture Protocol

#### 2.2.1. Blood Samples 

The majority of CTC cell lines have been established using 6 to 18 mL of blood. A blood volume >10 mL is a suitable source for CTC cultures. Furthermore, since the establishment of cell lines was found to be slightly easier from patients with a high CTC count (>30/mL), a CTC count >300 is preferable for the success of cultures. However, a high CTC count only does not guarantee the establishment of a permanent cell line. Furthermore, a conventional CTC count is generally assessed using EpCAM(+) cells. Since EpCAM(−) CTC have been overlooked, CTC counts are not accurate. The most recent study on CTC used a 96 mL sample obtained from leukapheresis for a cell culture [25]. Leukapheresis is used in clinical settings and is not harmful; however, more than 2 h is needed to obtain larger numbers of CTC from patients (Table 2 [9,10,11,12,13,14,15,16,17,18,19,20,21,22,23,24,25]).

#### 2.2.2. CTC Enrichment

An enrichment step for CTC is indispensable before cells are cultured. Viable CTC isolation may be divided into three types: antibody-based, physical property-based, and function-based approaches. The most successful enrichment method for long-term cultures is the negative selection method (the density gradient method or RosetteSep^®^). Although antibody-based approaches were employed in three studies, capture with antibodies against surface antigens is harmful, precluding its use for CTC cultures [9,15,20]. CTC analysis differs from CTC culture [28,29]. Our failure to establish a stem cell marker-selected CTC culture appeared to be partially attributed to the disadvantages associated with the positive selection method [27].

The use of a microfluidic device and nanotechnology-based method may be less harmful to CTC. One group used microfluidics-based immunomagnetic isolation [15,20] and another group used the CTC iChip [10,24]. Thus, microfluidic devices are also useful for long-term culture.

The RosetteSep^®^ antibody cocktail, which crosslinks unwanted cells in human whole blood to multiple red blood cells to form immune rosettes, has been used to enrich viable CTC and has contributed to the successful establishment of long-term cultures of CTC. Although negative selection will affect CTC purity, this is not critical for CTC cultures because leukocytes do not have a negative impact on cell cultures and are eliminated during the culture [30,31]. Nevertheless, since non-cancer cells may inhibit CTC growth, mimicking the in vivo tumor microenvironment as closely as possible may be important for the successful establishment of CTC cultures [21] (Table 2 [9,10,11,12,13,14,15,16,17,18,19,20,21,22,23,24,25]).

#### 2.2.3. Culture Conditions

Three-dimensional conditions with a low attachment plate or gel formation are generally employed for cultures. Cells have been cultured in spheroid or organoid 3D models. Yu et al. [10] previously suggested that non-adherent culture conditions were critical because CTC senesced after a few cell divisions in adherent monolayer cultures. They used ultra-low attachment culture dishes, while the other five conditions involving Matrigel and feeder cells failed [10]. However, attachment or Matrigel-coated plates were utilized for one-third of the cultures of CTC cell lines. Therefore, non-adherent conditions are not the gold standard (Table 2 [9,11,13,21,22,25]).

Hypoxic conditions have been recommended for stem cell cultures. Two-thirds of studies initially cultured cells under hypoxic conditions for several days. Hypoxic conditions generally use low attachment plates in the first step. However, one-third of studies used normoxic conditions [9,13,16,21,22,23,25]. Therefore, hypoxic conditions are also not always inevitable. Furthermore, several different hypoxic conditions (2, 4, and 8%) have been used during the establishment step for CTC cell lines, and the most suitable conditions have not yet been identified. Hypoxic conditions are used to maintain CTC and deplete normal cells. Cells are then transferred to the second stage using normoxic conditions [12,15] (Table 2).

#### 2.2.4. Culture Medium

Specific growth factor-enriched medium with or without fetal calf serum (FCS) has been used as the start medium. The culture medium is then changed to ordinal standard medium. The culture medium is important during the initial 2 weeks. The proliferation of most cells peaks after approximately 21 days. CTC with proliferative potential may then grow without special growth supplement, except for FCS (Table 2).

Epidermal growth factor (EGF) and basic fibroblast growth factor (FGF) are often listed in recipes for stem cell cultures. Recipes for serum-free CTC culture media mostly include proprietary B27 and N2 supplements, together with insulin, transferrin, and selenium. Y27632, a cell-permeable inhibitor of Rho-associated coiled-coil containing protein kinase, is also used. However, the optimal conditions for ex vivo CTC cultures have yet to be established.

#### 2.2.5. Growth Progression of Each CTC Cell Line

To appropriately culture CTC, it is important to understand the progression of CTC growth in each step. In this section, we focus on the growth progression of selected CTC cell lines.

1. CTC-1, CTC2, and CTC3

Zhang et al. [9] previously reported the culture of captured cells in a suspension using stem cell medium for the first week, and this was followed by a medium for epithelial cells. Cells were monitored for survival and growth over 28 days, and colonies were initiated from a single cell. Thirteen, seven, and eleven colonies were present by day 21 and were named CTC-1, CTC-2, and CTC-3, respectively.

2. CTC-MCC-41

In the case of CTC-MCC-41 cells, viable CTC were still observed after 4 days of culture in serum-reduced medium under hypoxic conditions. After 10 days, CTC started to proliferate and formed spheres. After a few weeks, the cell culture was switched to normoxic conditions. Single colonies from the CD45(−) population that appeared during the first week of the cell culture were transferred into a 24-well plate for further growth [12].

3. BHGc7 and BHGc10

BHGc cells initially grew as typical small spheroids that eventually showed the outgrowth of adherent tumor cells and shedding of apoptotic cell fragments [14]. Proliferating CD56-positive CTC cultures displayed loosely attached or compact spheroids as well as an adherent morphology from the same source [13]. Furthermore, all small cell lung cancer (SCLC) CTC cultures showed the spontaneous formation of large multicellular aggregates that increased in diameter to 1–2 mm, designated as tumor spheres, under regular cell culture conditions [16].

4. CTC-TJH-01

During an ex vivo culture of CTC-TJH-01 cells, contaminating blood cells and a small number of CTC underwent rapid cell death. The majority of surviving CTC adhered to the plate, but remained dormant throughout the initial two weeks, suggesting the low in vitro clonogenic potential of CTC. CTC started to proliferate after two weeks, and the culture was switched back to normoxic conditions. CTC slowly proliferated, formed clusters, and then exhibited sustained proliferation [15,20].

5. CTC-ITB-01

CTC-ITB-01 cells grew in parallel as adherent and non-adherent cell fractions with various cell sizes. Non-adherent cells grew from adherent cells. CTC showed an interchangeable adherent and non-adherent cell population similar to cancer stem cells [22].

6. UWG01CTC and UWG02CTC

In the case of UWG01CTC and UWG02CTC cells, viable, relatively pure cultures were observed within 3 weeks and rapidly expanded. UWG01CTC is adherent and requires trypsinization for passaging; however, a loose adherent spheroid phenotype was induced in a hypoxic environment and serum-free media. In contrast, UWG02CTC grew in long mucinous, loosely aggregated, and weakly adherent strands that only required gentle mechanical dissociation for passaging [23].

7. CTC-3

CTC-3 cells began to proliferate and form clusters after 14 days of culture [21]. However, other detailed descriptions were not provided.

#### 2.2.6. Timing of and Transfer Protocol for Each Cell Line

Several conditions were used for the expansion of CTC. However, suitable conditions depended on the characteristics of each cancer cell. Furthermore, the optimal timing and culture conditions for CTC expansion have not yet been established. Representative cases are described below.

1. CTC-1, CTC-2, and CTC-3

Adherent plates were used during the first week with growth factor-enriched stem cell culture medium. The medium was changed to EpiCult-C medium between 8 and 21 days. Standard medium with 10% FCS was then used for maintenance [9].

2. Mel-167, PEM-22, Mel-182, and PEM-78

CTC were cultured in 24-well ultra-low attachment plates for between 4 and 8 weeks. A 3D fibrin Matrigel culture was then used, and this was followed by a switch to an anchorage-independent culture [24].

3. CTC-MCC-41

CTC were cultured in 24-well non-adherent plates for the 1st week. Cells were then transferred to new 24-well non-adherent plates in the 2nd week and maintained in a T25 flask [19].

4. CTC-3

A Matrigel-coated plate was used in the first 2 weeks, and cells were then transferred to a normal 6-well plate [21].

## 3. Short-Term Culture

To establish approaches to long-term culture, short-term culture was reviewed. Information that will contribute to the establishment of long-term cultures of CTC was obtained (Table 3 and Table 4).

### 3.1. Overview of Short-Term Cultures

**Figure 1:**
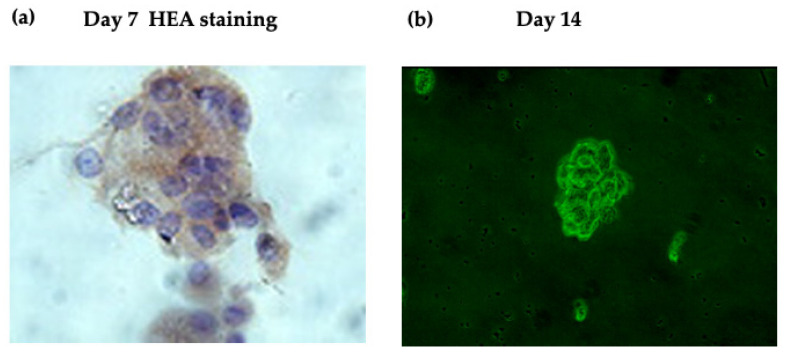
Representative images of the short-term CTC cell culture. (**a**) Cytospin image. Positive staining for Human Epithelial Antigen (HEA) was observed in cell clusters on day 7. (**b**) Phase contrast image of cell clusters on day 14 using a green filter [7].

**Figure 2:**
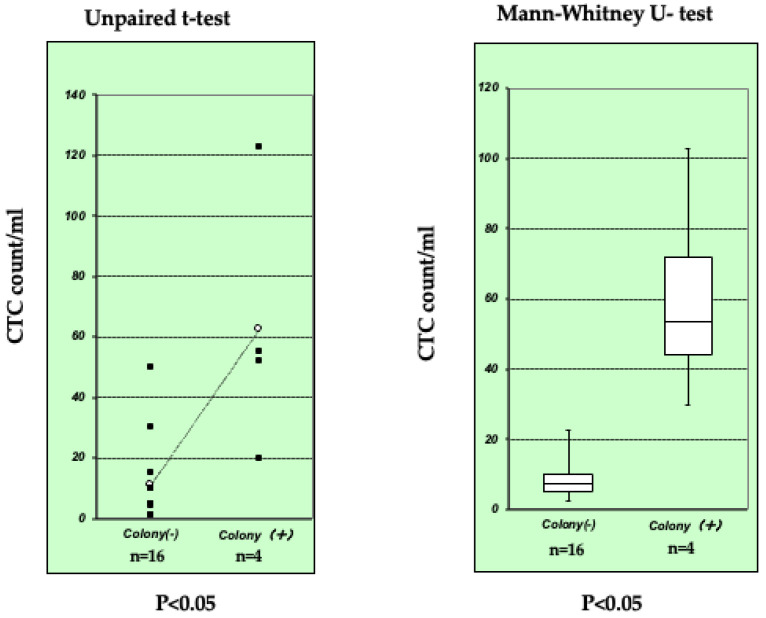
Colony formation by 4 out of 20 samples. The number of samples was too small for a definite evaluation; however, a high CTC count/mL (anti-HEA) was associated with colony formation (an unpaired *t*-test and the Mann–Whitney U test < 0.05) [7].

We successfully performed a short-term culture of CTC using a magnetic cell sorting system (MACS) with anti-Human Epithelial Antigen (HEA) 125 in 1999 [7]. We detected CTC (1 to 123 cells/mL) in 20/32 (63%) ESCC patients. Among 20 CTC-positive samples, 4 (20, 50, 52, and 123 CTC/mL) formed colonies after more than 14 days (Figure 1). However, we were unable to establish long-term cultures of these colonies. Although the number of samples was too small for a definite evaluation, a high CTC count was associated with colony formation (Figure 2). Approximately 30 studies on short-term cultures of CTC were subsequently published (Table 3 and Table 4 [7,32,33,34,35,36,37,38,39,40,41,42,43,44,45,46,47,48,49,50,51,52,53,54,55,56,57,58,59]). Ten of these studies were by the same research group and used the same method [35,36,37,38,39,40,44,45,47,51].

### 3.2. Enrichment of CTC

Similar to long-term cultures, most enrichment methods for cultures involve the negative selection method (the density gradient method or RosetteSep^®^). Few studies employed RBC cell lysis only. Under optimal conditions, the introduction of an RBC lysis step does not markedly affect the viability of the nucleated cell fraction [43]. Ten studies used MetaCell^®^ (filtration using a porous polycarbonate membrane) selection [35,36,37,38,39,40,44,45,47,51]. Four studies described the use of a microfluidic device [42,46,50,53]. Furthermore, diagnostic leukapheresis (DLA) and microfluidic enrichment obtained a large number of viable CTC from metastasized cancer patients. The average CTC yield per apheresis (mean volume, 59.5 mL) was 12,546 CTC [52].

Each method has its own advantages and limitations, and researchers have based the development of capture strategies on the specific aims of further CTC characterization studies [60] (Table 4).

1. Porous polycarbonate membrane

The MetaCell^®^ filtration device is a capillary action-driven, size-based separation method that isolates and enriches viable CTC from peripheral blood samples using a porous polycarbonate membrane (pores with a diameter of 8 μm) [35,36,37,38,39,40,44,45,47,51]. The culture with the device consists of a membrane (membrane fraction) and the bottom of a 6-well culture plate (invading fraction). CTC grow on the bottom of the culture plate after escaping the separating membrane through the pores. MetaCell^®^ detects cancer cells not only on the separating membrane, but also on the plastic bottom of the 6-well plate [39]. Membranes with captured cells may be transferred to culture plates [51]. The success rate for short-term cultures was high.

2. On-chip culture

Zhang et al. [42] described an on-chip culture of a microfluidic co-culture model. After 7 days of the on-chip culture, cells were released from the device by an incubation with collagenase. Cells were then flushed outside the device with media at a flow rate of 10 mL/h for 3 mL. Approximately 90% of cells were released from the device. The recovered population was reseeded on well plates and cultured for an additional 7–14 days. The on-chip culture successfully expanded CTC isolated from 14 out of 19 early-stage lung cancer patients.

### 3.3. Characteristics of Short-Term Cultures of CTC

Kapeleris et al. [54] suggested the identification of CTC as follows: (I) morphologically larger than background cells with intact nuclei; (II) a high nuclear–cytoplasmatic ratio (NC ratio); (III) positive for pan-cytokeratin; (IV) positive for DAPI; (V) negative for CD45; and (VI) cells larger than 14 μm. Kolostova et al. [40] also suggested defining CTC as cells exhibiting the following characteristics: (1) a nuclear size equal to or larger than 10 μm; (2) irregular nuclear contours; (3) the presence of a visible cytoplasm; (4) prominent nucleoli; (5) a high NC ratio; (6) changes in the NC ratio with the in vitro culture time; and (7) deformability/plasticity (growth through the membrane to the bottom and the formation of new colonies).

Regarding CD45, a significant population of “double positive” cells with hematopoietic and epithelial markers (CK+/CD45+) were detected in many patient samples [41]. Furthermore, a few reports suggested the existence of tumor–macrophage fusion cells (TMFs) in the patients’ blood [61,62]. Tumor-associated macrophages (TAMs) recruited to the stroma from circulating monocytes are required for tumor cell intravasation, migration, extravasation, and angiogenesis. TAMs have been hypothesized to fuse their membranes with those of tumor cells, forming tumor–macrophage hybrid cells [56].

Thus, most of the double positive cells may be the heterogeneous CTCs [41]. From the viewpoint of cell culture, CD45+ cells co-inhabited in the majority of the samples [57], and cultures grown in the presence of CD45+ cells exhibited higher growth potential ex vivo [56]. Furthermore, both macrophages and neutrophils associate with CTCs [56]. Thus, CD45+ cells may not always be excluded from the culture.

Khoo et al. [43] maintained CTC cultures for 2–8 weeks and noted that CTC with longer culture times exhibited more mesenchymal characteristics, with an increase in Vimentin and Fascin staining and the almost complete loss of epithelial characteristics. Malala et al. [49] attempted to characterize CTC and reported changes in their phenotype during in vitro cultivation.

The viability and morphology of expanded CTC were examined by LIVE/DEAD staining [55].

### 3.4. Culture Conditions and Medium

Few studies have employed low attachment plates and hypoxic conditions. Therefore, short-term cultures are generally aimed at clinical use, not long-term cultures. Therefore, they are routinely conducted under adherent and normoxic conditions. Furthermore, most studies used simple standard culture medium with FCS.

Tapered microwells are preferred over conventional cylindrical microwells for cluster formation. Cultures are sheltered within microwells with minimal disturbance during medium changes, which reduces shear stress and cell loss to allow for subsequent expansion [43]. This method takes advantage of a patient’s own white blood cells as co-culture components with CTC, and maintains their proximity within specialized microwells for maximum interaction. White blood cells from the same patient are feeder cells that promote CTC cluster formation [63].

Carmona-Ule et al. [57] suggested a culture medium supplemented with nanoemulsions composed of oleic acid and lipids for CTC growth (Table 4).

### 3.5. Success Rate and Culture Period

The success rate of cultures was previously reported to be more than 50%. The total success rate of MetaCell^®^ was 65%. Despite the leukapheresis approach, CTC from only a few patients have been successfully cultured. A success rate of 100% (12/12 samples) was achieved by Ficoll-Paque selection [56].

The typical cell culture period is <14 days and few cells may be cultured for more than 3 months. Typical spheres were shown to increase in size over time from 7–21 days [34]. Khoo et al. [43] reported that the proportion of CK+/CD45− cells significantly decreased after day 14 in most samples, and thus selected day 14 as the endpoint for culture phenotyping. This time point also correlated with the highest number of Ki67-positive clusters.

Carmona-Ule et al. [57] investigated the median progression time (100 days) and days of CTC in culture, and proposed 23.5 days as the best threshold for discriminating between groups (AUC = 0.65). Therefore, a cut-off of 23 days is considered to be optimal for CTC culture. Using this criterion, approximately 50% of studies achieved successful short-term cultures (Table 3 and Table 4).

Although proliferation was not observed due to the challenging ex vivo growth environment, the maintenance of CTC viability was guaranteed for at least two months [46]. Eliasova et al. [51] reported that some isolated CTC grew in vitro for up to 6 months as a standard cell culture; however, the exact number of cases was not described.

### 3.6. Growth Patterns of Short-Term Cultures of CTC

#### 3.6.1. Cell Distribution

Khoo et al. [43] showed that cytokeratin-positive (CK+) cells localized at the center of tapered microwells and were surrounded by CD45+ cells. The majority of large cells within and outside the microwells expressed CD68, which was suggestive of macrophages.

#### 3.6.2. Presence of Undetectable CTC

Khoo et al. [43,63] demonstrated that some blood samples that did not initially contain detectable CK+ CTCs in culture were positive on day 14. This may have been due to proliferation of very few CTC with heightened survival characteristics. Therefore, initial CTC counts before culture do not always reflect the potential for cluster formation. Samples with an undetectable level of CTC before culture may still form clusters.

#### 3.6.3. Morphology

The morphological spheroid construct typically consists of three different presentable types: large-sized, cohesive round-shaped spheroids; small-sized cohesive irregular or round spheroids; and discohesive “grape-like” spheroids [55]. Some samples presented cells growing both in suspension and adherence [57].

In the MetaCell^®^ system, CTC grew through the membrane on the bottom of the well after 14 days in an in vitro culture exhibiting two different phenotypes, epithelial-like and stem cell-like. Stem cell-like cells formed visible cell clumps. Cells growing on the bottom showed a very plastic morphology [40]. CTC increased in size and became elongated during in vitro growth, which altered the NC ratio [44].

#### 3.6.4. Unexpected Effects of Blood Cells

Non-adherent blood cells provide a cushion layer that keeps CTC in suspension [61]. CTC have been shown to attach to the top of adherent leukocyte cells and expand [56]. 

### 3.7. Timing of the Culture Process

After 1 week of a cell culture under hypoxic conditions, cells are generally switched to standard cell culture conditions. When 85% confluence is reached, growing cells are transferred to an ultra-low attachment 24-well plate [57]. However, the optimal timing for the transfer of cultured cells to the next step currently remains unclear.

### 3.8. Difference between CTC Cell Lines and Short-Term Cultures 

Regarding the establishment of CTC cell lines, the majority of cultured CTC lack the ability to proliferate. Therefore, 2 months is a sufficient time period to assess whether CTC exhibit the ability to continuously proliferate. Future studies that compare CTC lines with CTC that stop proliferating after a short-term culture will facilitate the development of optimal molecular conditions for the permanent survival and growth of CTC [22].

## 4. Our Experience

Cell growth depends on the proliferative potential of each cell. Among 21 conventional esophageal cancer cell lines, the time after the initiation of the primary culture to the second culture varied from 2.5 weeks to 48 weeks, depending on cell growth patterns, such as the adhesion-dependent type, spheroid type, and mixed type. Adaptability to a new environment is also important [2].

We previously reported the maintenance of several cancer cells in protein-free medium without other supplements for 2 years [3]. These cell lines exhibited the ability to grow without exogenous growth factors; however, another study showed that lipids, insulin, and transferrin were essential factors for the proliferation of some carcinoma cell lines [28,57] 

Based on our experience and previous findings, we preliminarily performed a culture of CTC collected from patients with prostate cancer and renal cell carcinoma. We recently developed a CTC capture system [64]. We first attempted an on-chip culture using gel formation or a culture of released CTC under a high flow. We successfully established a short-term culture of CTC (20 out of 27 samples), but not a long-term culture. 

We also used VIVANT-CELL^®^-pot (porous polycarbonate membrane selection) with Lymphoprep or negative selection using RosetteSep^®^ CD45, CD36, or CD56. A short-term culture of CTC was successful for 16 out of 27 samples; however, expansion was very challenging to overcome. One reason is that we did not obtain a high number of viable CTC from patients because most had already received several courses of chemotherapy. The most important issue is that we have not yet established a specific protocol for individual carcinoma cells.

**Figure 3:**
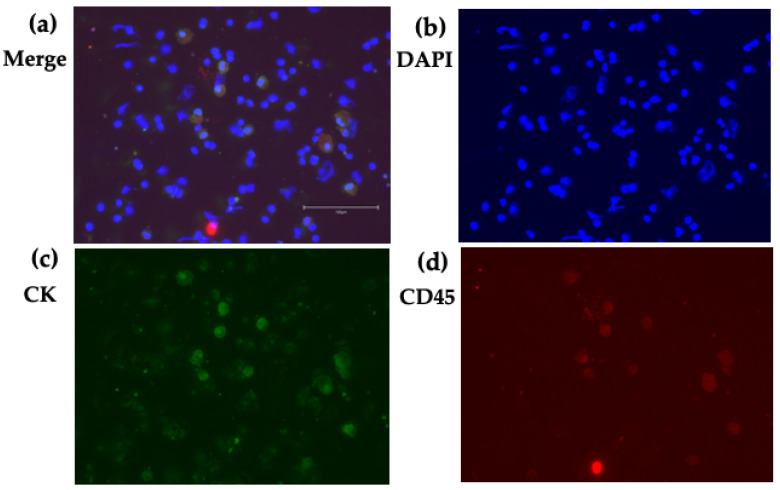
CK/CD45 double positive cells. Cells obtained from the short-term (35 days) CTC culture. Cytospin image: (**a**) Merge, (**b**) DAPI, (**c**) CK, and (**d**) CD45. The scale bar shows 150 μm. Some cells express both CK and CD45.

**Figure 4:**
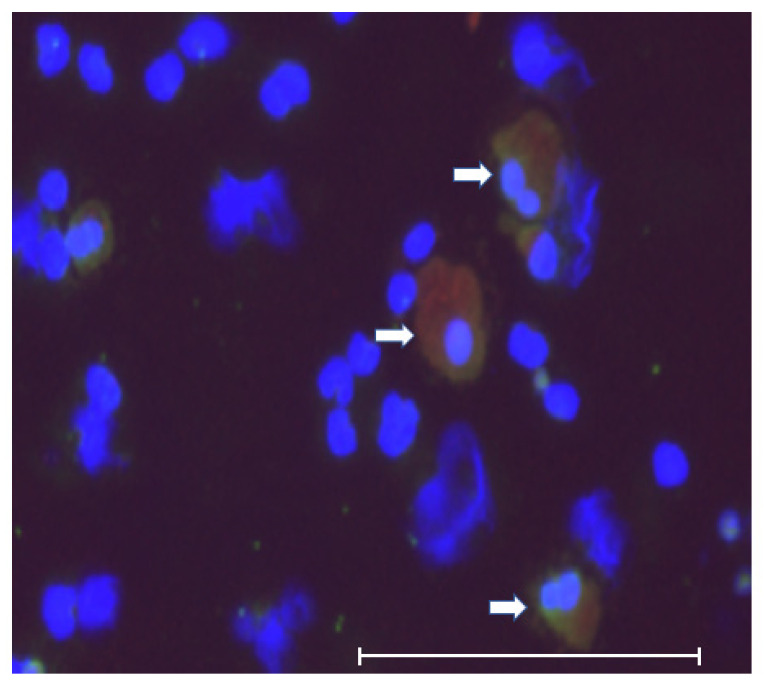
Morphology of double positive cells. These cells were large, polymorphic, and polynuclear, which suggested that they were tumor–macrophage fusion cells (TMFs), macrophage–tumor cell fusion cells (MTFs), or cancer-associated macrophage-like (CAMLs) cells. The scale bar shows 150 μm. White arrows indicate CK/CD45 double positive cells.

Regarding double positive (CK+/CD45+) cells, we also detected these cells in the short-term culture of CTC. Some of these cells were large, polymorphic in shape and polynuclear (Figure 3 and Figure 4). These morphological features suggested that these were tumor–macrophage fusion cells (TMFs), macrophage–tumor cell fusion cells (MTFs), or cancer-associated macrophage-like (CAMLs) cells [62].

In several trials, we developed three cell lines with the spontaneous immortalization of lymphocytes from different patients under conditions involving several growth supplements, such as Y-27632 and Z-VAD-fmk. We previously reported the incidental establishment of B-cell cell lines (ITSM), derived from EB virus spontaneous activation during an ordinary cell culture from pseudomyxoma peritonei [65]. The long-term use of growth supplements exerted unexpected effects on the culture. Wang et al. [28] also suggested the application of exogenous factors, but was unable to increase the success rate of CTC cultures and may result in undesired complications. Therefore, careful management is needed during cell cultures. These findings suggest the use of growth supplements for a set time period only.

## 5. Critical Points

CTC cell lines have unique intermediate characteristics between primary tumors and distant metastasis. CTC are not simple travelers in the bloodstream. Although most CTC are apoptotic and sensitive to stress, a few viable CTC survived.

The CTC count of samples is the most important factor for the successful establishment of CTC cell lines. A CTC count of >100 cells/mL blood is favorable. Leukapheresis may be the most powerful method for enriching CTC; however, its success rate is low.

There is currently no evidence to support the contribution of CD45+ cells to the success of long-term culture. However, a reconsideration of the parameters for CTC isolation is warranted [56].

It is important to note that because optimal CTC culture conditions have yet to be defined, current research relies on stem cell culture methods that ensure maximal CTC expansion. Each protocol is designed based on a researcher’s own expertise and empirical results, and not by a rationale based on the biological features of CTC, which are presently not defined [28].

Moreover, activated lymphocyte proliferation is transient, ending with programmed activation-induced cell death. Macrophages are the only cell type among peripheral blood mononuclear cells that are capable of surviving for weeks in culture. However, they are also a terminally differentiated cell type that is incapable of ex vivo proliferation [28].

Other key technical limitations include the maintenance of the CTC phenotype and the composition of a stable population in culture. Previous studies suggested that normal human mesenchymal stem cells are prone to genomic changes and subsequent malignant transformation in long-term cultures [66].

Therefore, the further optimization of CTC culture conditions and a more detailed understanding of the differences between non-adherent CTC cell lines and adherent cell lines as well as CTC cultures and patient samples is required [67].

## 6. Protocol Currently Recommended for CTC Cell Lines

As described above, the recommended protocol for CTC cell lines is as follows (Table 5). With careful optimization, these culture conditions may be applied to successful long-term cultures.

## 7. Conclusions

A careful review of previous studies suggested that CTC in long- and short-term cultures have different characteristics. Not only the culture method, but also the characteristics of CTC themselves affect the success of establishing CTC cell lines.

In the circulation, CTC from any type of cancer encounter the same environment, which differs from their solid tissue location. Only cells with specific characteristics, such as stem cell-like cells, may survive in the bloodstream. CTC without these characteristics are protected by surrounding cells, such as leukocytes and platelets. However, it currently remains unclear whether only CTC with stem cell features are suitable long-term culture.

Low attachment plates and hypoxic conditions may be the ideal conditions for the initial culture steps. A successful culture medium contains several growth factors and anti-apoptotic supplements. However, as described above, cancer cells have already acquired growth and survival autonomy. Therefore, CTC cultured in the presence of growth factors and small molecule inhibitors may not be ideal for modeling cancer and stromal interactions. The most important factors are the timing of CTC transfer to the next step and the careful management of medium changes.

Short-term culture is the initial approach to long-term culture; however, prolonged protocols sometimes deviate from the main expansive route for establishing CTC cell lines. The success of short-term cultures is not always affected by a high number of CTC; however, long-term cultures generally require high numbers of CTC. Therefore, the culture method may only prolong cell survival; it does not affect permanent proliferative activity. The most important issue is obtaining CTC that are suitable for long-term culture; however, a method to identify these CTC has not yet been established.

There is currently no gold standard method. The selection of an optimal method for each sample may be achieved through careful management and continued efforts. Extensive trials are needed to establish long-term cultures of CTC. Without these efforts, CTC that are suitable for expansion will not be identified.

## Figures and Tables

**Table 1:** Summary of CTC cell lines.

Cell Line	Type of Cancer	Establishing Rate	Culture Period or Passage	Doubling Time	Xenograft	Specific Characteristics	References
CTC-1, CTC-2, CTC-3	Breast cancer	3/38	Several passages?	N/A	Yes (all 3 CTC lines)	EpCAM(−)	Zang et al. (2013) [9]
BRx33, BRx07, BRx68, BRx50, BRx42, BRx61	Breast cancer	6/36. Several cell lines from the same patient at different times	>6–12 months	3 days to 3 weeks	Yes (3 out of 5 CTC cells lines)	ER(+), PIK3CA, TP53, KRAS, FGFR2	Yu et al. (2014) [10]
MSK-PCa5	Prostate cancer	1/17	Passage was performed weekly at a 1:3 ratio	1 week	Yes	CK(+), AR(+),High PSA, PTEN(−), RB(−)	Gao et al. (2014) [11]
CTC-MCC-41	Colon cancer	1/50 (CTC-positive patients)	>16 months	20 h	Yes	EpCAM(+), Snail, ALDH1, CD133,	Cayrefourcq et al. (2015) [12]
BHGc7, BHGc10	Small cell lung cancer	2/30	>4 months. Long-term culture was confirmed by two follow-up studies	N/A	Yes	Carbonic anhydrase (CAIX)	Hamilton et al. (2015) [13], (2016) [14]
N/A (one line was named CTC-TJH-01 by Que [20])	Lung adenocarcinoma	2/35	>6 months	N/A	N/A		Wang et al. (2016) [15]
BHGc16, BHGc26, UHGc5	Small cell lung cancer	N/A	N/A	N/A	N/A	CD133, CD24, SOX-2	Klameth et al. (2017) [16]
CTC44, CTC45 (both established from the same patient)	Colorectal cancer	3/4. Several cell lines from the same patient at different times	>20 passages	N/A	Yes	CD133, CD26, ALDH1A1, CD44	Grillet et al. (2017) [17]
BRx82, BRx142	Breast cancer	N/A	N/A	101.2 and 59.7 h	N/A	Able to cryopreserve	Sandlin et al. (2017) [18]
CTC-MCC-41.4, 41.5A. 41.5B. 41.5C, 41.5D. 41.5E, 41.5F, 41.5G (established from the same patient)	Colon cancer	N/A. Several cell lines from the same patient at different times (8 cell lines)	N/A	N/A	N/A	ALDH1, CD44, panCD66, EpCAM	Soler et al. (2018) [19]
CTC-TJH-01	Non-small cell lung cancer	1/89	24 months	N/A	Yes	CXCL5, CD44, ALDH1	Que et al. (2019) [20]
CTC-3	Breast cancer	1/16	>2 years	N/A	Yes	CD44	Zhao et al. (2019) [21]
CTC-ITB-01	Breast cancer	1/50	>4 years	N/A	N/A	E-Cadherin, EpCAM, K19, CD24, Twist 1	Koch et al. (2020) [22]
UWG01CTC, UWG02CTC	Gastroesophageal cancer	2/23	>12 months	N/A	Yes	EpCAM(−), CD56, (UWG01CTC) or EpCAM(+), CK(+), CD44, E-Cadherin	Brungs et al. (2020) [23]
Mel-167, PEM-22, Mel-182, PEM-78	Melanoma	4/37. Several cell lines from the same patient at different times (Mel 182-1, Mel 182-2)	N/A	N/A	Yes	BRAF-mutant NG-2, MLANA	Hong et al. (2021) [24]
EMC-Pca-41	Prostate cancer	1/40	>1 year, 10 passages	N/A	N/A	TMPRSS2-ERG fusion, loss of PTEN	Mout et al. (2021) [25]

N/A: not available.

**Table 2:** Culture protocols of CTC cell lines.

Cell Line	Blood Volume	CTC Count/mL	CTC Enrichment and Isolation	Culture Type	Environment	Culturing Conditions	Culture Medium	References
CTC-1, CTC-2, CTC-3	20 to 45 mL	Undetectable by CellSearch	FACS (CD45, ALDH1, EpCAM)	2D. single cell to colony formation	5% CO_2_. Normoxic conditions	Adherent (1–8 days), medium change (8–21 days). The EpCAM(−)/ALDH1(+)/CD45(−) population was transferred to 24- or 6-well plates	Stem cell culture medium. Insulin, hydrocortisone, B-27, EGF, FGF-2, (1–8 days). EpiCult-C medium supplemented with 10% FCS (8–21 days). DMEM/F12 supplemented with 10% FCS (from day 22)	Zang et al. (2013) [9]
BRx33, BRx07, BRx68, BRx50, BRx42, BRx61	6 to 18 mL	3–3000/6 mL	CTC iChip	3D Spheroid	4% O_2_	Ultra-low attachment plate. Medium changes were performed under a microscope	Serum-free, EGF, FGF, B-27	Yu et al. (2014) [10]
MSK-PCa5	8 mL	>100 count/8 mL	RosetteSep CD45-depleted Cocktail	3D Organoid. Start to grow as spheroids after 10 days	N/A	Matrigel	DMEM/F12, EGF, R-spondin 1, Noggin, FGF10, FGF2, DHT, Nicotinamide Acros, A83-01, SB202190, Y-27632, B27, N-Acetyl-L-cysteine, Glutamax, HEPES, Primocin	Gao et al. (2014) [11]
CTC-MCC-41	10 mL	302 count/7.5 mL	RosetteSep CD45-depleted Cocktail	3D Spheroid	Initial environment: 2% O_2_. Maintenance: 5% CO_2_, Normoxic conditions	24-well non-adherent plate. T25 flask (maintenance)	Initial medium: Stem cell culture medium, DMEM/HamF12 2% FCS, insulin, N2 component, EGF, L-Glutamine, FGF2. Second medium: RPMI1640 EGF, FGF2, insulin-transferrin-selenium supplement, L-Glutamine. Maintenance: N/A	Cayrefourcq et al. (2015) [12]
BHGc7, BHGc10	N/A	N/A	Ficoll-Hypaque density gradient	3D and 2D	Normoxic conditions	12-well adherent plate. Normoxic conditions	Initial medium: Serum-free, RPMI-1640, insulin, IGF-1, transferrin, selenite, Maintenance: RPMI-1640, 10% FCS	Hamilton et al. (2015) [13] (2016) [14]
N/A (one line was named as CTC-TJH-01 in Que Z paper [20])	2 mL	130 count/2 mL	Microfluidics-based immunomagnetic isolation. EpCAM coated and EGFR coated immunomagnetic microbeads	N/A	3% O_2_, 5% CO_2_ (1–14 days). 5% CO_2_ Normoxic conditions	96-well non-adherent plate.	Initial medium: RPMI1640, EGF, FGF, B27. Maintenance: RPMI-1640, 10% FCS	Wang et al. (2016) [15]
BHGc16, BHGc26, UHGc5	N/A	N/A	N/A	3D and 2D	Normoxic conditions	N/A	Initial medium: N/A. Maintenance: RPMI-1640, 10% FCS	Klameth et al. (2017) [16]
CTC44, CTC45 (both established from same patient)	8–10 mL	N/A	RosetteSep CD45-depleted Cocktail	3D	N/A	Ultra-low attachment 24-well plate	DMEM/F12, 2% FCS, L-Glutamine, N2 supplement, EGF, FGF2	Grillet et al. (2017) [17]
BRx82, BRx142	N/A	N/A	N/A	3D	N/A	6-well ultra-low adhesion plate	Initial medium: N/A, Maintenance: RPMI1640 EGF, FGF2, B-27	Sandlin et al. (2017) [18]
CTC-MCC-41.4, 41.5A. 41.5B. 41.5C, 41.5D. 41.5E, 41.5F, 41.5G, (established from same patient)	10 mL	286/7.5 mL–3278/7.5 mL	RosetteSep CD45-depleted Cocktail	3D	N/A	24-well non-adherent plate (1st week). New 24-well non-adherent plate (2nd week). T25 flask (maintenance)	RPMI 1640, EGF, FGF-2, insulin-transferrin-selenium supplement, L-Glutamine	Soler et al. (2018) [19]
CTC-TJH-01	5 mL	130 count	A mixture of EpCAM and EGFR coated immunomagnetic microbeads in microfluidic Herringbone-Chip	2D	N/A	Non-adherent plate	Initial medium: RPMI1640, EGF, FGF, B27. Maintenance: RPMI-1640, 10% FCS	Que et al. (2019) [20]
CTC-3	6 mL	N/A	RosetteSep CD45-depleted Cocktail	2D	Normoxic conditions, 5% CO_2_	6-well Matrigel-coated plate (2 weeks), normal 6-well plate (medium change every 2–3 days). Final culture in a T25 flask	DMEM/RPMI1640, 10% FCS, EGF, FGF, Nu-Serum, L-Glutamine	Zhao et al. (2019) [21]
CTC-ITB-01	7.5 mL	1547/mL	RosetteSep CD45-depleted Cocktail	3D and 2D	Normoxic conditions, 5% CO_2_ (1–14 days)	96-well normal plate, then transferred to a 12-well culture dish	RPMI1640 10% FCS, L-Glutamine, insulin-transferrin selenium-A, FGF2, EGF, hydrocortisone, cholera toxin	Koch et al. (2020) [22]
UWG01CTC, UWG02CTC	15 mL	3/mL (UWG01CTC), 109/mL (UWG02CTC)	RosetteSep CD36-depleted Cocktail	2D or 3D	Normoxic or hypoxic conditions	24-well ultra-low attachment plate	DMEM/F12, EGF, FGF, N2 supplement (normoxic conditions) or DMEM/F12, 10% FCS (hypoxic conditions)	Brungs et al. (2020) [23]
Mel-167, PEM-22, Mel-182, PEM-78	10 mL	N/A	CTC iChip	3D	Hypoxia, 5% CO_2_, 4% O_2_	24-well ultra-low attachment plate (4–8 weeks). Use of a 3D fibrin Matrigel culture. Finally, a switch to an anchorage-independent culture	RPMI 1640, EGF, FGF2, B-27, Heparin, Y-27632	Hong et al. (2021) [24]
EMC-PCa-41	5 L	5312/96 mL	Leukapheresis, RosetteSep CD45-depleted Cocktail	3D Organoid	Normoxic conditions	24-well plate Matrigel droplets. Organoids were collected and resuspended in a new plate	Initial medium: Prostate growth medium (PGM) or adjusted prostate cancer organoid medium (APCOM). Maintenance: AdMEM/F12	Mout et al. (2021) [25]

EGF: Epidermal growth factor, FGF: Fibroblast growth factor.

**Table 3:** Summary of short-term cultures.

Research Group	Cancer Type	Establishing Rate	CTC Count	CTC Detection	Culture Period	Additional Information
Makino et al. (1999) [7]	Gastrointestinal cancer	4/20	1–123/mL by cytospin	HEA(+), CK(+)	14 days	High CTC were more likely to form spheroids
Paris et al. (2009) [32]	Prostate cancer	5/8	150–740/mL (CRPC), 0–100/mL (CSPC)	Pan CK(+), CD45(−), EpCAM	1 week to 3 months	
Lu et al. (2010) [33]	Breast cancer	10/10	18–256/mL	EpCAM, Pan CK, CD45	1–33 days	Collagen adhesion matrix (CAM)
Pizonet al. (2013) [34]	Breast cancer	31/39	1700–9360/mL	EpCAM(+)	28 days	Number of spheres was 0–29/mL
Bobek et al. (2014) [35]	Pancreatic cancer	16/24	NA	Nuclear size, NC ratio, Irregularity, CK7, DAPI, CD45(−)	>14 days	
Bobek et al. (2014) [36]	Esophageal cancer	27/43	NA	Nuclear size, NC ratio, Irregularity, CK18, DAPI, CD45(−)	<14 days	
Bobek et al. (2014) [37]	Pleural Mesothelioma	4/5	NA	Nuclear size, NC ratio, Irregularity, MPF, OPN, DAPI, CD45(−)	10–14 days	
Ceganet et al. (2014) [38]	Urinary bladder cancer	25/39	1–50/8 mL	Nuclear size, NC ratio, Irregularity, CK18, DAPI, CD45(−)	14 days	
Kolostova et al. (2014) [39]	Urothelial tumors	NA	NA	Nuclear size, NC ratio, Irregularity, CK7, DAPI	10 to 14 days	
Kolostova et al. (2014) [40]	Prostate cancer	18 (proliferative capacity)/28 (CTC-positive)/55 (total cases)	NA	Nuclear size, NC ratio, Irregularity, CK7, DAPI	14 days	
Sheng et al. (2014) [41]	Pancreatic cancer	0/12	0–7/mL	CK(+), CD45(−), DAPI	no proliferation	Capture and release
Zhang et al. (2014) [42]	Lung cancer	14/19	1 to 11/mL	CK(+), CD45(−)	NA	p53 mutation did not always match between primary tissue and CTC
Khoo et al. (2015) [43]	Breast cancer	7/18 (early stage)	NA	CK(+), CD45(−)	2–8 weeks	
Kolostova et al. (2015) [44]	Ovarian cancer	77/118	NA	Nuclear size, NC ratio, Irregularity, CK, DAPI, CD45(−)	3–14 days	CA125-positive
Kolostova et al. (2015) [45]	Gynecological cancer	3/3	NA	Nuclear size, NC ratio, Irregularity, CK7, DAPI, CD45(−), Muc1	3–10 days	
Chen et al. (2016) [46]	Colorectal cancer	NA	0–1000/mL	DAPI, CK20(+), CD45(−)	10 days to 2 months	Maintenance for up to 2 months, but no growth was observed. p53 mutation, APC
Kolostova et al. (2016) [47]	Gastric cancer	13/22	NA	Nuclear size, NC ratio, Irregularity, CK18, 19, DAPI, CD45(−)	>14 days	
Kulasinghe et al. (2016) [48]	Head and neck cancer	7/25	1–15 CTC/5 m by cytospin	EpCAM(+), CK(+) CD45(−), morphologically larger than background cells. High NC ratio	21 days to 63 days. 3D is longer than 2D	Successfully obtained higher CTC counts. HPV-positive
Malala et al. (2016) [49]	Colon cancer	7/7	5/mL (CD133(+)), 29/mL (CK20(+))	CK20(+), CD45(−)	14 days	The characterization of CTC revealed changes in their phenotypes during their cultivation in vitro
Zhang et al. (2016) [50]	Hepatocellular carcinoma	31/36 (>100 μm defined as spheroids)	1–42/2 mL	ASGPR, CK(+), CD45(−)	>7 days	Capture and release. ASGPR(+)
Eliasova et al. (2017) [51]	Colorectal cancer	81/98	NA	Nuclear size, NC ratio, Irregularity, CK, DAPI, CD45(−)	3–5 days, some were able to grow for 6 months	
Lambros et al. (2018) [52]	Prostate cancer	2/14	12546/7.5 mL	DAPI, CK(+), CD45(−)	>4–6 weeks	Two organoids from the same patient
Franken et al. (2019) [53]	Breast cancer	2/8 (without cryopreserved samples), 3/9 (cryopreserved samples)	1–2913 CTC/7.5 mL	CK(+), CD45(−)	>3 months	Successfully cultured from cryopreserved samples
Kapeleris et al. (2020) [54]	Non-small cell lung cancer	9/70	0–385/7.5 mL	DAPI, CK(+), CD45(−), high NC ratio, larger than background cells	20 to 50 days	Culturability was not affected by an increased number of CTC. EGFR mutation
Lee et al. (2020) [55]	Small cell lung cancer	18/22	8–277/mL	CK(+), CD45(−), EpCAM, TTF-1, Synaptophysin	2 to 6 weeks	BCC, Platelet lysate
Xiao et al. (2020) [56]	Breast cancer	12/12	NA	CK5, CK8, Mammaglobin	>30 days (6 cases), <30 days (6 cases)	Presence of CD45(+) cells exhibited higher growth potential ex vivo. Does not exclude CD45(+) cells
Carmona-Ule et al. (2021) [57]	Breast cancer	36/50	0−1000/7.5 mL	EpCAM, Pan CK, CD45	>23 days (up to 291 days, mean 8 weeks)	Nanoemulsions support CTC. Some cells express CD45 (+), CD36(+)
Hu et al. (2021) [58]	Hepatocellular carcinoma	55 (spheroid)/60 (CTC)/106 (total)	NA	ASGPR/CPS1, DAPI, EpCAM, CD45(−)	12–14 days	Beta-catenin (+), a spheroid was defined as a 3D cell structure >100 μm. ASGPR(+)
Yang et al. (2021) [59]	Gastrointestinal cancer	13 (colony)/38 (viable cell)/81 (total)	NA	hTERT	4 weeks	J2 feeder cell-coated plate

HEA: Human epithelial antigen. CRPC: Castration-resistant prostate cancer. CSPC: Castration-sensitive prostate cancer. ASGPR: Asialoglycoprotein receptor 1.

**Table 4:** Protocol for the short-term culture.

Authors	Blood	CTC Enrichment and Isolation	Culture Type	Environment	Culture Condition	Culture Medium
Makino et al. (1999) [7]	30 mL	Magnetic cell sorting system (MACS) anti-HEA125 (EpCAM)	2D semi-spheroid	37 °C, 5% CO_2_	60 mm culture dish	RPMI1640/F12, 10% FCS
Paris et al. (2009) [32]	3 mL	Ficoll-Paque	2D	N/A	16-well chamber slide coated with Collagen adhesion matrix (CAM)	DMEM/RPMI1640, 10% FCS, Nu-serum, L-glutamine
Lu et al. (2010) [33]	0.5 mL	Ficoll density gradient centrifugation, Collagen adhesion matrix (CAM) capture	2D	37 °C, 5% CO_2_	CAM-coated 96-well microtiter plate	RPMI1640, 10% FCS, Nu-serum, L-glutamine
Pizon et al. (2013) [34]	1 mL	Erythrocyte lysis	3D	37 °C, 5% CO_2_	25 cm^2^ culture flask	RPMI1640, low FCS, L-glutamine, EGF, Insulin, Hydrocortisone
Bobek et al. (2014) [35,36,37]	8 mL	MetaCell: filtration using a porous polycarbonate membrane (pores with a diameter of 8 μm)	2D	37 °C, 5% CO_2_	On the membrane and bottom of the 6-well culture plate	RPMI1640, 10% FCS
Cegan et al. (2014) [38]	8 mL	MetaCell: filtration using a porous polycarbonate membrane (pores with a diameter of 8 μm)	2D	37 °C, 5% CO_2_	On the membrane and bottom of the 6-well culture plate	RPMI1640, 10% FCS
Kolostova et al. (2014) [39,40,44,45,47]	8 mL	MetaCell: filtration using a porous polycarbonate membrane (pores with a diameter of 8 μm)	2D	37 °C, 5% CO_2_	On the membrane and bottom of the 6-well culture plate	RPMI1640, 10% FCS
Sheng et al. (2014) [41]	5–10 mL	GEM chip	2D	37 °C, 5% CO_2_	60 mm culture dish	DMEM, 10% FCS
Zhang et al. (2014) [42]	5 mL	Microfluidic CTC capture chip	3D	37 °C, 7.5% CO_2_	On chip co-culture (fibroblast) 7 days. Well plate for 7 days	RPMI complete medium, 10% FCS
Khoo et al. (2015) [43]	10 mL	RBC lysis	3D spheroid	37 °C, 5% CO_2_, 1% O_2_. Normoxic conditions (maintenance)	Tapered microwell (14 days). 3D (Geltrex) ultra-low adhesive dish	DMEM, 10% FCS. Then, DMEM/F12, reduced serum
Chen et al. (2016) [46]	2 mL	Lipid bilayer-coated microfluidic system (CMx platform) EpCAM, gentle sweep of air foam	2D and 3D	N/A	Normal attachment culture dish or ultra-low attachment plate	DMEM medium, EGF, FGF, Insulin, B-27
Kulasinghe et al. (2016) [48]	10 mL	RosetteSep CD45-depleted Cocktail	2D and 3D	2% O_2_, 5% CO_2_	96-well standard microplates, Spheroid microplates, Happy Cell^®^ hydrogel for 3D expansion	DMEM/F12, EGF, R-Spondin, Noggin, FGF10, FGF2, Nicotinamide, A83-01, SB202190, Y-27632, B27, N-Acetyl-L-cysteine, Glutamax, Hepes, Primocin
Malala et al. (2016) [49]	5 mL	Ficoll-Paque	3D sphere	N/A	Culture dishes	DMEM/F12, Heparin, EGF, FGF, BSA
Zhang et al. (2016) [50]	2 mL	Microfluidic chip, Capture and release	3D Gel	37 °C	Matrigel 24-well plate	DMEM: Matrigel 1:1, 10% FCS
Eliasova et al. (2017) [51]	8 mL	MetaCell: filtration using a porous polycarbonate membrane (pores with a diameter of 8 μm)	2D	37 °C, 5% CO_2_	On the membrane and bottom of the 6-well culture plate	RPMI1640, 10% FCS
Lambros et al. (2018) [52]	40 to 100 mL	Apheresis, EasySep EpCAM-positive selection	3D organoid	N/A	Ultra-low attachment surface-coated microplates	Growth factor-reduced Matrigel
Franken et al. (2019) [53]	3.41 L	Diagnostic Leukapheresis (DLA), microfluidic Parsortix system	3D	5% CO_2_, 4% O_2_	Low attachment plate	Tumor sphere medium, RPMI1640, B27, EGF, FGF,
Kapeleris et al. (2020) [54]	10 mL	RosetteSep CD45-depleted Cocktail, RBC lysis	N/A	1–2% O_2_	96-well standard microplates	DMEM/F12, EGF, R-Spondin, Noggin, FGF10, FGF2, Nicotinamide, A83-01, SB202190, Y-27632, B27, N-Acetyl-L-cysteine, Glutamax, Hepes, Primocin, 10% FCS
Lee et al. (2020) [55]	7.5 mL	RosetteSep	2D and 3D	N/A	96-well binary colloid crystal (BCC) substrate	DMEM/F12, EGF, bFGF, B27, Platelet lysate
Xiao et al. (2020) [56]	7.5 mL	Ficoll-Paque	2D	37 °C	Culture dishes	DMED/F12 B27, EGF, FGF, Heparin, Y-27632, Adenine, L-Glutamine
Carmona-Ule et al. (2021) [57]	7.5 mL	RosetteSep CD56-depleted Cocktail	3D	37 °C, 5% CO_2,_ 1–2% O_2_ (1 week). Then, normoxic conditions	96-well ultra-low attachment plate. Then, 24-well ultralow attachment. 25T flask (maintenance)	MammoCult media, Progesterone, Beta-estradiol, Heparin, Hydrocortisone, UltraGRO, B-27, bFGF, EGF, Nanoemulsions
Hu et al. (2021) [58]	5 mL	Ficoll-Paque, CD45 depletion by magnetic separation	3D Gel	37 °C, 5% CO_2_	24-well plate	DMEM, Matrigel, 10% FCS
Yang et al. (2021) [59]	5 mL	RBC lysis	2D	37 °C, 5% CO_2_	J2 feeder cell-coated plate	DMEM, 5% FCS, L-glutamine, F12 nutrient mix, Hydrocortisone, EGF, Insulin, Cholera toxin Y-27632

**Table 5:** Protocol currently recommended for CTC cell lines.

**1 First step**
(1) Blood sample volume >10 mL
(2) Negative selection for CTC enrichment (RosetteSep^®^, etc.) or Ficoll-Paque density gradient centrifugation
(3) Hypoxic conditions within 10 days (7 days is recommended)
(4) Low attachment plate or gel formation
(5) Growth factors (such as EGF, FGF, and B-27) and anti-apoptotic supplements (including Y-27632) with FCS
**2 Option**
(1) Preservation of CD45(+) cells.
(2) Normoxic conditions and attachment plates dependent on the cell type
(3) Microfluidic device or nanotechnology-based method
(4) Serum-free medium
(5) Co-culture with normal leukocytes or fibroblasts
(6) Culture medium changes under the microscopic ministering of cell clusters
(7) Exclusion criteria
(i) the use of chemotherapy and/or antibody-based therapy in the past 4 weeks
(ii) the use of radiotherapy in the past 2 weeks
**3 Second step (after 7 to 14 days of the initial culture)**
(1) Transfer to normoxic conditions
(2) Standard culture medium
**4 Third step (after several weeks)**
(1) No standard method
(2) Select specific conditions depending on each cell
(3) Careful management
**5 Critical steps**
(1) Medium changes at 200 μL/min or less every 72 h [64]
(2) In the case of a highly proliferative culture, an increased frequency of medium changes
(3) To prevent the excessive stimulation of normal blood cells, the use of anti-apoptotic supplements for long periods is not recommended

## Data Availability

Most recent data which described in this review were preliminary data and did not report any data.

## Abbreviations

N/A: not available; FACS: fluorescent-activated cell sorting; SCLC: small cell lung cancer; NSCLC: non-small cell lung cancer; 2D: two-dimensional culture; 3D: three-dimensional culture. HEA: Human epithelial antigen.
